# Adulteration detection in cactus seed oil: Integrating analytical chemistry and machine learning approaches

**DOI:** 10.1016/j.crfs.2025.100986

**Published:** 2025-01-22

**Authors:** Said El Harkaoui, Cristina Ortiz Cruz, Aaron Roggenland, Micha Schneider, Sascha Rohn, Stephan Drusch, Bertrand Matthäus

**Affiliations:** a Max Rubner-Institut, Federal Research Institute for Nutrition and Food, Department for Safety and Quality of Cereals, Schützenberg 12, 32756, Detmold, Germany; b Department of Food Chemistry and Analysis, Institute of Food Technology and Food Chemistry, Technische Universität Berlin, Berlin, Germany; c Department of Food Technology and Food Material Science, Institute of Food Technology and Food Chemistry, Technische Universität Berlin, Berlin, Germany; d Max Rubner-Institut, Federal Research Institute for Nutrition and Food, Zentralabteilung, Haid-und-Neu-Str. 9, 76131, Karlsruhe, Germany; e Max Rubner-Institut, Federal Research Institute for Nutrition and Food, Zentralabteilung, Schützenberg 12, 32756, Detmold, Germany; f Johann Heinrich von Thünen Institute - Federal Research Institute for Rural Areas, Forestry and Fisheries, Bundesallee 50, 38116, Braunschweig, Germany; g BMEL Project KIDA, AI consultancy, Germany

**Keywords:** Cactus seed oil, Authenticity, Machine learning, Conditional generative adversarial network, Monte-Carlo, Random Forest, Neural network

## Abstract

Economically motivated adulteration threatens both consumer rights and market integrity, particularly with high-value cold-pressed oils like cactus seed oil (CO). This study proposes a machine learning model that integrates analytical measurements, data simulations, and classification techniques to detect adulteration of CO with refined sunflower oil (SO) and determine the detectable limit of adulteration without measuring a huge number of different mixtures. First, pure CO and SO samples were analyzed for their fatty acid, triacylglycerol, and tocochromanol content using HPLC or GC. The resulting oil composition data served as the foundation for further simulations. Monte Carlo (MC) simulations outperformed Conditional Tabular Generative Adversarial Networks (CTGAN) in simulating realistic oil compositions, with MC yielding lower Kullback-Leibler Divergence values compared to CTGAN. The MC-simulated data were then used to simulate larger datasets, a critical step for training and testing two classification models: Random Forest (RF) and Neural Networks (NN), as robust training cannot be achieved with small sample sizes. Both models achieved good classification accuracies, with RF achieving higher accuracy than NN, reaching 94% on simulated datasets and 90% on real-world samples with detectable adulteration levels as low as 1%. RF also offers better interpretability and is computational less demanding as compared to NN which makes it advantageous for authenticity verification in this study. Therefore, combining MC simulation with RF as a robust method for detecting CO adulteration is proposed. The proposed method, coded in Python and available as open-source, offers a flexible framework for continuous adaptation with new data.

## Highlights

• Machine learning methodology developed to detect cactus oil adulteration.
• Monte Carlo outperformed CTGAN in simulating oil compositions.
• Random Forest model achieved 90% accuracy in detecting cactus oil adulteration.
• Adulteration levels as low as 1% of refined sunflower oil were detectable.
• Open source Python code available for replication.

## Graphical abstract

**Figure:**
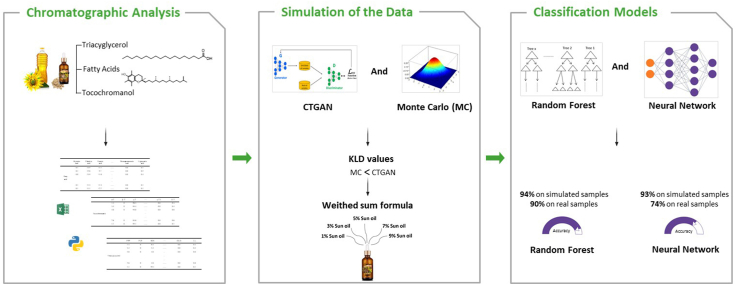

## 1 Introduction

Economically motivated adulteration poses a significant threat to consumer rights and market integrity, with cold-pressed vegetable oils being particularly susceptible to fraudulent practices (Dou et al., 2023; Yuan et al., 2020). Among these oils, expensive varieties such as cactus seed oil (CO) are more prone to adulteration. Detecting and preventing such fraudulent practices is essential not only for maintaining consumer trust and safeguarding fair trade practices but also for protecting public health and supporting robust regulatory frameworks.

Initially, CO is extracted from the seeds of the resilient cactus plants of the genus *Opuntia*. This oil provides a rich profile of essential fatty acids, antioxidants, and vitamins, making it a potential source of edible oil, particularly in arid regions where the cactus thrives (Nounah et al., 2024; Chbani et al., 2023). CO is characterized by its high levels of linoleic acid and abundant gamma-tocopherol content (Nounah et al., 2024). Additionally, its polyphenol composition includes significant amounts of vanillin, syringaldehyde, and furaldehyde (Chbani et al., 2020). The olfactometric analysis further identifies hexanal, 2-methyl propanal, acetaldehyde, acetic acid, acetoin, and 2,3-butanedione as the most abundant aroma-active compounds, contributing to CO's characteristic flavor (Nounah et al., 2020). These components among others contribute to CO's diverse biological activities, which include *in vivo* and *in vitro* antioxidant effects, as well as antimicrobial, antidiabetic, lipid-lowering, anticancer, anti-inflammatory, and anti-ulcer properties (Barba et al., 2017; Ramadan and Tamer, 2021; Chbani et al., 2023; Al-Naqeb et al., 2021). Despite its edible nature, the primary application of CO currently lies in the cosmetic industry, where it is valued for its moisturizing, anti-aging, and skin-nourishing properties (Ramadan and Tamer, 2021; Chbani et al., 2023). The oil is marketed for its hydration potential, ability to improve skin elasticity, and to reduce skin redness and pigmentation. It also contains vitamin K1, which helps reduce dark circles and spider veins (Ramadan and Tamer, 2021).

In Morocco, CO production is a labor-intensive process, predominantly carried out by cooperatives in regions where the *Opuntia* cactus (mainly *Opuntia ficus-indica*) thrives. The extraction of oil from the small seeds requires significant quantities of raw material, contributing to the oil's high pricing (approx. 600 €/L in bulk) which underscores the economic motivation for adulteration (Chbani et al., 2020). The adulteration of CO can not only affect the health-promoting properties of the oil but also erode consumer's trust in CO and other products sold by cooperatives, highlighting the importance of ensuring its authenticity. Recent studies that have characterized CO from the perspectives of extraction, chemical composition, and potential applications, concluded that investigation of its potential adulteration remains an important, yet underexplored, research area (Nounah et al., 2024; Chbani et al., 2020, 2023).

Adulteration of high-value oils often involves diluting the expensive oil with inexpensive oils rather than outright substitution (Azadmard-Damirchi and Torbati, 2015). Commonly available and inexpensive refined oils are often chosen as potential adulterants (Dou et al., 2023). Adulterants that closely mimic organoleptic properties, such as smell and color of the expensive oil are preferred. Additionally, the market availability and the chemical composition of the adulterant are also considered. Based on this fact, refined sunflower oil (SO), with its similar visual appearance and fatty acid composition, is a likely candidate for adulterating CO. For detecting adulteration, one approach would be to focuses on analyzing a specific marker, such as trans fatty acids or stigmastadienes, which are often employed in detecting virgin-refined oil mixtures (Aued-Pimentel et al., 2013; Jabeur et al., 2014). While this approach have proven useful in certain cases, they are not always sufficient especially for lower levels of adulteration (Jabeur et al., 2014). Another key limitation of marker-based detection is that counterfeiters can specifically target these markers to avoid detection. An alternative approach involves analyzing multiple aspects of the oil's chemical composition, such as its fatty acids, triacylglycerols, and tocopherols. This multi-parameter approach strengthens detection by making it harder for counterfeiters to manipulate the oil's composition to avoid detection. Fatty acids, triacylglycerols, and tocopherols are frequently analyzed using chromatographic techniques such as gas chromatography (GC) or high-performance liquid chromatography (HPLC), which are both reliable and widely accepted methods in quality control laboratories. Chromatography techniques are appreciated in terms of robustness, sensitivity, precision, and accuracy, which are key parameters also useful for authenticity studies (Shi et al., 2022; Xing et al., 2019; Ilić et al., 2022; Esteki et al., 2018).

Supporting analytical approaches with chemometrics has proven to be a powerful tool for detecting adulteration in vegetable oils (Esteki et al., 2018). Chemometrics applies mathematics and statistical methods to process acquired data, particularly useful when results cannot be attained through the analysis of a single chemical marker but require the generation of multivariate data sets (i.e., analysis of the entire fatty acid composition) (Kamal and Karoui, 2015; Sudhakar et al., 2023). Distinguishing pure oils from adulterated ones presents a multivariate classification task, where classification models are employed to discriminate between pure and adulterated oils. Various classification models in combination with chromatographical techniques have been applied to detect adulteration in vegetable oils, each demonstrating its performance in different scenarios (Sudhakar et al., 2023; Zhang et al., 2017a). However, the size of the dataset is critical for building a robust classification model, and insufficient data may lead to overfitting, reducing the model's reliability (Qiu et al., 2018; Zhang et al., 2022; Shorten and Khoshgoftaar, 2019).

To generate an adequate dataset for training classification models, traditional approaches involve preparing and analysing a large number of pure oil samples and mixtures with varying levels of adulteration. This process, which includes physically combining and shaking the samples, is time-consuming and resource-intensive, presenting a significant challenge. Further, this challenge underscores the need for innovative approaches that optimize data generation and enhance model robustness without relying solely on extensive experimental work. To address this, data augmentation techniques have been introduced. Data augmentation methods involves the creation of new data by either slightly modifying original data or simulating new synthetic data based on artificial intelligence and statistical modeling (Gracia Moisés et al., 2023; Shorten and Khoshgoftaar, 2019; Georgouli et al., 2018; Wang et al., 2024). By incorporating simulated data, the dataset size can be expanded, enabling the exploration of a broader range of oil compositions.

To address the issue of CO adulteration, this study aimed at developing a machine learning model that integrates analytical measurements, data simulations, and classification techniques to detect CO adulterated with SO and determine the minimum detectable level of adulteration, without the need to analyze an extensive number of physical mixtures. First, oil samples, including pure CO and SO, were analyzed for their fatty acid, triacylglycerols, and tocochromanol using liquid and gas chromatography techniques (HPLC and GC). This analysis provided a database of oil compositions, serving as the foundation for subsequent simulations. Specifically, two simulation techniques, Monte Carlo simulation (MC), and Conditional Tabular Generative Adversarial Networks (CTGAN), were then employed to simulate oil compositions. By comparing the generated data from both models to the real samples, the model that produced the most accurate simulations should be identified. Using the selected model, oil mixtures were simulated by varying the proportions of CO and SO. The generated data from these simulations was then used to train two classification models: Random Forest (RF) and Neural Network (NN). At the end the trained models were evaluated for their accuracy in detecting adulteration on real-world samples. To ensure the model's long-term validity and applicability beyond the current dataset, the entire methodology was coded in Python and will be made publicly available. This approach enables continuous updates, allowing the model to remain a reliable resource for detecting CO adulteration as its market evolves.

## 2 Material and methods

**Fig. 1:**
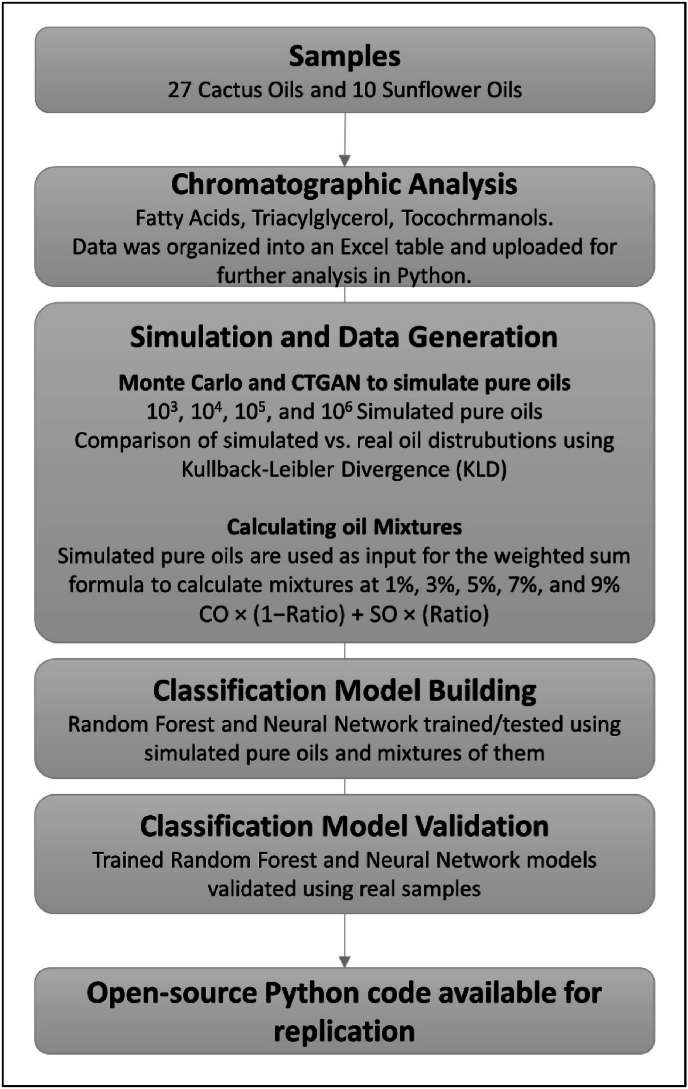
Flow chart summarizing the methodology.

To provide a comprehensive overview of the study, a workflow summary is presented in Fig. 1.

The following subsections describe each part of the methodology in detail, starting with the sample collection.

### 2.1 Material

The cold-pressed CO samples, in total 27 (labeled CO1 … CO27), were purchased from partner cooperatives across various regions of Morocco. The fruits of the cactus (*Opuntia ficus-indica*) were peeled, and the seeds were manually collected, washed, and sun-dried within the cooperatives. Oil extraction was performed using a screw press. The cooperatives were carefully selected from different locations in Morocco to ensure diverse provenance, and the oil quality was guaranteed by the cooperatives themselves. SO samples, in total 10 (labeled SO1 … SO10), were purchased from local Moroccan markets, representing the two main brands available in the Moroccan market (Lesieur Cristal Group (HuilOR) and Huileries du Souss Belhassan (Zohor)). Adulterated oils were prepared in brown glass bottles at concentrations of 1%, 3%, 5%, 7%, and 9%, with each sample weighing 3 g. For practical reasons related to the nature of the oil matrix, mass-based measurements were chosen when preparing the mixtures. The oils were precisely measured using an analytical balance (AUW220, SHIMADZU, Japan) with an accuracy of 0.1 mg. The samples were then subjected to overnight shaking using a Shaker Mixer (Turbula T2F, Switzerland) for preparing a homogeneous blend. The prepared samples were stored at −18 °C until further analysis.

### 2.2 Determination of the fatty acid composition

Gas chromatography was employed to determine the fatty acid (FA) composition, following standard methods DGF C-VI 10a (00) and C-VI 11d (19) (DGF, 2021). Initially, a drop of oil was dissolved in 1 mL *n*-heptane (for liquid chromatography; LiChrosolv®, Supelco®, Merck KGaA, Darmstadt, Germany) and mixed with 50 μL sodium methylate (30% solution in methanol; Merck KGaA, Darmstadt, Germany). The mixture was agitated for 1 min at room temperature (22 °C), and then 100 μL of Millipore water was added. After centrifuging at 1550×*g* for 5 min, the lower aqueous phase was removed, and 50 μL 1M hydrochloric acid (min. 25.0%, p.a; Chemsolute®) was added along with some drops of the indicator methyl orange (ACS reagent, dye content 85%; Merck KGaA, Darmstadt, Germany). Following brief mixing, the lower aqueous phase was removed. Next, 20 mg sodium hydrogen sulphate (EMSURE® ACS, ISO, Reag. Ph Eur, Merck KGaA, Darmstadt, Germany) were added, and after centrifuging at 1550×*g* for 5 min, the top *n*-heptane phase was transferred to a vial and injected into an HP5890 gas chromatograph (Agilent Technologies Deutschland GmbH, Waldbronn, Germany). The chromatograph was equipped with a CP-Sil 88 capillary column (100 m × 0.25 mm × 0.25 μm; Agilent Technologies Deutschland GmbH, Waldbronn, Germany). The temperature was gradually increased from 150 °C to 250 °C at a rate of 1.5 °C/min and was maintained at 250 °C for 5 min. The injector and detector were set to 260 °C, with the carrier gas (H_2_) flow rate at 1.7 mL/min and a 1:50 split ratio. The detector was operated using 40 mL/min hydrogen, 400 mL/min air, and 40 mL/min nitrogen and had an injection volume of 1 μL. The fatty acid methyl esters (FAME) were identified by comparing their retention times with a standard mix (Supelco®37 Component FAME Mix; Merck KGaA, Darmstadt, Germany) and their composition was quantified as a percentage of the total FA.

### 2.3 Determination of the triacylglycerol composition

The triacylglycerol (TAG) composition was assessed using gas chromatography following standard method DGF C-VI 14 (08) (DGF, 2021). The analysis was conducted on an Agilent 6890 gas chromatograph equipped with a flame ionization detector (Agilent Technologies Deutschland GmbH, Waldbronn, Germany). Fifty milligrams of oil were dissolved in 10 mL isooctane (EMSURE®, ACS, Reag. Ph Eur; Merck KGaA, Darmstadt, Germany), and 1 μL of this solution was injected into an RTX®65 TG column (30 m × 0.25 mm × 0.1 μm; Restek Corp., Bellefonte, PA, USA). The oven temperature was held at 300 °C for 1 min, then increased from 300 °C to 360 °C at a rate of 2 °C/min and held at 360 °C for 10 min. The injector and detector were set to 370 °C, and the carrier gas (H2) flow rate was 1 mL/min with a 1:40 split ratio. The detector operated using 40 mL/min hydrogen, 450 mL/min air, and 45 mL/min nitrogen. According to the DGF method, TAG were identified by comparing their retention times with those of sesame oil, which were previously established for TAG composition analysis. Commercial TAG standards, PaOlPa, PaOlOl, StOlOl, OlOlOl (99%, Merck KGaA, Darmstadt, Germany), were used for the identification process, as well ('Pa' represents palmitic acid, 'St' represents stearic acid, and 'Ol' represents oleic acid). The composition of each TAG was calculated as the percentage of its peak area relative to the total peak area of all detected peaks.

### 2.4 Determination of the tocochromanol composition

The tocochromanol content and composition were determined following standard method DGF F-II 4a (00) (DGF, 2021). Initially, 150 mg of oil were dissolved in 1 mL of *n*-heptane (for liquid chromatography LiChrosolv®, Supelco®, Merck KGaA, Darmstadt, Germany) and filtered using a 1.0 μm filter (Whatmann®, Maidstone, UK) followed by a 0.45 μm filter (Restek Corp., Bellefonte, Pennsylvania, USA). The filtered solution was then transferred into a vial and afterwards injected into the HPLC-FLD. The HPLC system configuration comprised a pump (L-7100 LaChrom Elite, Merck KGaA, Darmstadt, Germany), an autosampler (L-2200 LaChrom Elite, Merck KGaA, Darmstadt, Germany), a fluorescence detector (L-2485 LaChrom Elite, Merck KGaA, Darmstadt, Germany), and the interface box (Knauer Interface Box IF2, Berlin, Germany). To perform an isocratic separation, a diol phase column (25 cm × 4 mm × 5 μm, LiChroCART® 250-4, Merck KGaA, Darmstadt, Germany) was utilized. The mobile phase comprised *n*-heptane/tert.butyl methyl ether (for liquid chromatography LiChrosolv®, Supelco®, Merck KGaA, Darmstadt, Germany) at a flow rate of 1.3 mL/min. The injection volume for all samples was 20 μL, and the analysis time took 66 min. The fluorescence detector was set to an excitation wavelength of 295 nm and an emission wavelength of 330 nm. The tocochromanols were identified using α-, β-, δ-, and γ-tocopherol reference standards (chromatographic purity 97.6–99.6%, Merck KGaA, Darmstadt, Germany) and quantified through external calibration with standard solutions (0.25–40 μg/mL). The tocochromanol content was quantified in mg/kg of oil, and these values were used to calculate the proportion of each tocochromanol relative to the total tocochromanol content (%).

### 2.5 Statistical methods

The analysis of FA, TAG, and tocochromanols of the samples were performed in two analytical replicates, with the results reported as means and standard deviations, to ensure that the measurements are reliable and not affected by external factors. For the subsequent sections on simulations and classification models, the mean values were used as robust estimates of the chemical values. Attributes below the limit of quantification or those not detected in one of the samples were assigned a value of zero.

#### 2.5.1 Simulation models and data generation

In order to capture the higher variety of oils and to increase the database for more robust classification models, data were generated using two different methods: MC and CTGAN. The decision to use both CTGAN and MC simulations was made to compare a traditional statistical method with a deep learning approach in the generation of synthetic data adapted to the aims of the study. MC methods provide a well-proven approach for simulating data based on distributions. In contrast, CTGAN provides a more flexible and powerful tool with a little assumption for capturing complex relationships within the data. To our knowledge CTGAN has not been used before in the area of authenticity testing. By using both methods, their respective strengths should be evaluated and the most effective technique for our study should be identified to ensure that the generated samples closely approximate the statistical properties of the original data. The simulation process was conducted in two parts: first, the pure oils, CO and SO, were simulated using both MC and CTGAN. The simulated dataset that more closely matched the estimated real (observed) distribution was then used to calculate the synthetic mixtures, which were subsequently applied in the classification task.

**Monte Carlo (MC):** are a group of algorithms with different applications, such as modeling real-world scenarios and estimating possible outcomes. In the context of artificial intelligence, MC methods can be used to generate artificial data by randomly sampling data points from the probabilistic distribution of real data (Metropolis and Ulam, 1949). Assuming that each provided real CO and SO samples (observed samples) follow a normal distribution, the mean values for each of the features for CO and SO were computed, respectively, yielding a vector with 37 mean values for CO and SO. The mean values provide the central point of the distribution and ensure that the generated samples will be allocated around it. In order to capture the relations between the features, the covariance matrix, which provides the variance of the features (diagonal of the matrix), and the covariance between pairs of features (off-diagonal values), was computed. The covariance matrixes for SO and CO are reported in Fig. S1 and Fig. S2, respectively. With the covariance matrix, the variability and the relationships among the features for CO and SO in the real data is preserved in the generated samples. Thus, the samples were drawn from a multivariate normal distribution with 37 mean values of the features and a 37 × 37 covariance matrix capturing the relationship between the 37 features for SO and CO separately.

**Conditional Tabular Generative Adversarial Networks model (CTGAN):** is a generative deep-learning model that can learn the distribution of tabular data (without distribution assumption) and generate new data points out of that distribution (Goodfellow et al., 2014). These networks consist of two opposite networks, the generator and the discriminator. The generator is trained on generating data similar to the original data and the discriminator tries to differentiate between fake data points generated by the generator and real data. Both networks get trained simultaneously, while only the discriminator sees the real data. The discriminator then tries to distinguish between fake data and real data. After every training of an epoch, the weights of both networks get adjusted with backpropagation. The loss function of the generator determines how wrong the discriminator labeled the data and the loss function of the discriminator determines how right the data got classified. CTGAN, as part of a broader family of deep learning-based synthetic data generators, is particularly suited for handling single-table tabular data (Xu et al., 2019). In this study, the CTGAN model was trained using 27 samples of CO and 10 samples of SO, with training conducted over various numbers of epochs (100, 500, 1,000, 4,000, 10,000, and 50,000).

For comparing MC and CTGAN, between 1,000 and 1,000,000 synthetic data points (per class) were generated using both methods. The accuracy of the simulations was evaluated using the Kullback-Leibler Divergence (KLD) (Kullback and Leibler, 1951). KLD offers a holistic metric by measuring the divergence between two probability distributions, providing a comprehensive assessment of the model's overall performance. It is defined in the formula below (Formula 1):

(1) $$D_{KL}\left(N_{0}\;||N_{1}\right)=\frac{1}{2}\left(trace\left(\Sigma_{1}^{-1}\;\Sigma_{0}\right)+{\left(\mu_{1}-\mu_{0}\right)}^{T}\Sigma_{1}^{-1}\left(\mu_{1}-\mu_{0}\right)-k+\log\left(\frac{\det\;\Sigma_{1}}{\det\;\Sigma_{0}}\right)\right)$$

$\Sigma_{1}$ is the non-singular covariance matrix of the observed data with dimension k

$\Sigma_{0}$ is the non-singular covariance matrix of the simulated data with dimension k

$\mu_{1}$ are the mean values of the observed data

$\mu_{0}$ are the mean values of the simulated data

The KLD is a positive value that is 0, when the two distributions are identical. The larger the KLD value, the more distant are the two distributions. Since the covariance matrices for CO and SO are singular (i.e., not invertible), small noise was added to these matrices to make them invertible for the KLD computation.

According to the KLD values, only the simulations from the more accurate model (MC or CTGAN) will be used to calculate mixtures of CO adulterated with SO at varying ratios 99:1%, 97:3%, 95:5%, 93:7%, and 91:9% using the weighted sum formula (Formula 2).

(2) CalculatedMixture=CO×(1−Ratio)+SO×(Ratio)

where CO is the value of a certain feature of cactus oil and SO the value of the same feature of sunflower oil and “Ratio” the level of sunflower oil adulteration between 1% and 9%.

#### 2.5.2 Classification models

Random Forest and Neural Networks were selected as the two widely used classification models, with simulated pure oils and their mixtures serving as the training data. The two models were further tested on simulated data and on small real-world dataset.

**Random Forest (RF):** is a supervised machine learning algorithm built from an ensemble of Decision Trees. The trees classify the data set samples using a flowchart structure with yes/no questions that divide the tree nodes in a binary fashion. Usually, RF is preferred to Decision Trees because it is more robust and less prone to overfitting. In each tree and at each node, the features of the data are evaluated using a metric to determine which feature best splits the node into purer subsets i.e., nodes with the maximum possible number of samples belonging to a single class. The final predicted class for a sample is the class with the highest average probability among all the trees (Ho, 1995; Breiman, 2001). To enhance performance, RF can be fine-tuned through hyperparameter optimization. In this study, the following hyperparameters were tuned:

• Maximal depth the trees can reach (max_depth).
• How data will be selected to build the trees (bootrstrap).
• Number of features to consider for the best split (max_features).
• Minimum number of samples required to split a node (min_samples_split).
• Minimum number of samples to consider a node leaf or final node (min_samples_leaf).
• Number of trees of the RF (n_estimators).

For hyperparameter tuning the tool GridSearchCV from Scikit-learn was used. This approach takes a set or grid of hyperparameters and tests every possible combination of them to find out the best values. GridSearchCV also performs cross-validation, allowing the model being trained/tested on different subsets of the data. The output of the search is a set of hyperparameters for which the model achieved the highest performance in terms of accuracy.

**Neural Network (NN):** is a machine learning models inspired by the structure and function of the biological brain. The fundamental unit of a NN is the artificial neuron, which processes and transmits information. Each neuron receives input signals, combines them into a single output value, and applies a activation function to determine the neuron's response. The influence of each incoming signal is determined by a weight, which is adjusted during the training process. Neurons are organized into layers, typically with no connections within the same layer. Information flows through the NN in one direction, from the first layer (input layer), through multiple hidden layers, to the final output layer (Bishop, 2006; Schmidhuber, 2015). In this study, a fully connected NN was used. The network's input size matches the number of features (37), and the output layer corresponds to the number of classification classes (7). The following hyperparameters were tuned:

• Number of hidden layers: the hidden layers are the layers between input and output layer.
• Dimension for each hidden layer: number of neurons in each hidden layer.
• Batch size: number of data points fit to the NN in each iteration during the training.

The hyperparameter tuning was performed with the optuna framework. The framework tunes the hyperparameters based on the target metric, for which the accuracy was chosen. Optuna creates a trial with different values of hyperparameters. The values can be specified as a certain value space so the values are not chosen randomly. The defined values are reported as footnote in Table S2. Then the optuna randomly searches the value spaces of the hyperparameters to find the combination of hyperparameters with the best accuracy.

#### 2.5.3 Coding and libraries

Machine learning models for oil simulation and classification were built using Python, with specific libraries addressing various methodological needs. MC simulations were performed with numpy (Harris et al., 2020), CTGAN with the CTGAN library (Xu et al., 2019), RF with Scikit-learn (Pedregosa et al., 2011), and the NN using the Pytorch Lightning library (Paszke et al., 2019). Hyperparameter tuning for NN was done using optuna (Akiba et al., 2019). Exploratory data analysis (EDA) was conducted in Jupyter notebooks using numpy, pandas, matplotlib, and PyTorch. GPU support was utilized for NN training, while CPUs were used for RF. The open-source script is freely available on the following GitHub (https://github.com/kida4bmel/oil-adulteration). The increase of dataset size impacts model performance and processing time, so that the availability of resources is a relevant factor when selecting the settings.

## 3 Results and discussion

### 3.1 Chemical composition of the samples

Regarding the chemical composition, the CO and SO samples were analyzed in terms of FA, TAG, and tocochromanol. The complete results are reported in the following data publications for CO (El Harkaoui et al., 2024a, 2024c, 2024b) and in the supplementary data B for SO.

For CO, the primary FAs identified were palmitic acid (Pl, 11.4–12.9%), oleic acid (Ol, 13.0–23.0%), and linoleic acid (Li, 54.6–65.4%), which together comprise approximately 90% of CO's total FA composition. These values are consistent with previous studies on Moroccan CO, such as those by Taoufik et al. (2015) which reported similar ranges for the major FA: palmitic acid (11.75–12.3%), oleic acid (18.2–22.6%), and linoleic acid (60.2–64.6%). The stable and characteristic FA profile of CO is further supported by multiple studies on Moroccan samples (El Harkaoui et al., 2023; Ettalibi et al., 2020, 2021; Gharby et al., 2015, 2021; Nounah et al., 2021, 2024). This consistency suggests a stable and characteristic FA profile for CO, which remains largely unaffected by processing.

For SO, the FA composition revealed palmitic acid (5.6–6.9%), oleic acid (28.5–32.7%), and linoleic acid (54.6–59.1%), in the same predominance order as CO. Similar to CO, these three FAs account for approximately 90% of SO's total FA composition. The results for SO are within the ranges specified for SO by the Codex Alimentarius Standard for named Vegetable Oils (Codex, 1999). The major FA in both oils, linoleic, oleic, palmitic, and stearic acids constitute the main monomers of TAGs such as PaLiLi, LiLiLi, and LiLiOl. FA which accounts for 95–98% of the oil composition (Yara-Varón et al., 2017), serve as a chemical fingerprint that remains relatively stable even after processing and storage, making them reliable indicators of oil authenticity.

Both CO and SO contain α-tocopherol and γ-tocopherol as their predominant tocopherol isomers, with differences in their distribution. In CO, γ-tocopherol typically makes up the majority, ranging from 92 to 97%, while α-tocopherol is found in smaller amounts (up to 4%). In SO, α-tocopherol tends to be more prominent, around 96–97%, with γ-tocopherol present in lower proportions (up to 4%). Detailed tocochromanol composition for both oils can be found in the following data publication (El Harkaoui et al., 2024b) and in the supplementary data B for SO. The tocopherol profile for the analyzed CO seed oils is consistent with previously published results on Moroccan samples, showing similar patterns in tocopherol isomer dominance, although with slight variations in the reported ranges (Nounah et al., 2021, 2024; El Harkaoui et al., 2023; Gharby et al., 2015, 2020, 2021; Taoufik et al., 2015). For SO, the tocopherol content aligns with the ranges specified by the Codex Alimentarius Standard for named Vegetable Oils (Codex, 1999). While tocopherols offer additional insights for authenticity detection, their profiles can be influenced by factors such as light exposure, thermal degradation, mold growth, and refining processes. This sensitivity underscores the need for caution when relying solely on tocopherols for adulteration detection (Jee, 2002). The examination of multiple variables together with machine learning models (which leverages multiple features for decision-making), should enhance robustness and make it more difficult for counterfeiters to manipulate the composition of the oil to avoid detection.

### 3.2 Simulation of the data

Two simulation models, MC and CTGAN, were employed to simulate data, with the objective of comparing their effectiveness in producing samples that closely mirror the distribution of the original data. While box plots are commonly used to evaluate simulation results by visually comparing the distribution of raw and simulated data (Zhang et al., 2022; Cui et al., 2024), they are less practical in the context of multivariate simulations due to the large number of variables involved, which would require a high number of box plots. To address this, the KLD for multivariate normal distributions was utilized. The results are shown in Table 1.

**Table 1:** Kullback-Leibler Divergence values for data sets of different sizes simulated with Monte Carlo (MC) and Conditional Tabular Generative Adversarial Network (CTGAN) [values in 10^3^].

Simulation per oil		MC	CTGAN by Epochs					
			100	500	1,000	4,000	10,000	50,000
1,000	CO	3.1	974.3	972.7	898.4	272.5	187.2	105.1
	SO	1.4	2,531	2,266.2	1,717.4	595.6	209.0	76.8
10,000	CO	2.5	991.5	949.9	898.6	277.2	192.7	89.5
	SO	1.2	2,482.9	2,303.5	1,676.2	603.6	207.1	68.2
100,000	CO	2.3	997.7	952.3	895.1	277.8	194.3	93.1
	SO	1.3	2,470.9	2,282.6	1,681.7	460.7	208.5	68.3
1,000,000	CO	2.3	1,000.7	950.2	899.8	272.3	195.3	92.9
	SO	1.3	2,471	2,279.1	1,683.9	512.4	207.8	68.2

The MC method demonstrated consistent performance across all tested data set sizes, maintaining rather low KLD values for each sample size, especially from 10,000 samples onwards. For CO, KLD values ranged between 2.3 and 3.1, while for SO, the values remained between 1.2 and 1.4. An improvement is noticeable from 1,000 to 10,000 samples reducing KLD value from 3.1 to 2.5 and 1.4 to 1.2, respectively. In contrast, the KLD values of CTGAN were several times higher and were heavily influenced by the number of training epochs. At lower epochs (100–1,000), CTGAN's KLD values were significantly higher than those of MC, indicating a less accurate simulation of the original data. For example, with 1,000 simulations, KLD values for SO were as high as 2,531 at 100 epochs. However, as the number of epochs increased, CTGAN's ability to mimic the original data improved slightly. At 50,000 epochs, CTGAN produced the lowest KLD values across all scenarios, with 89.5 for CO and 68.2 for SO at 10,000 simulations. But even with 50,000 epochs, all KLD are several times higher than for MC. The size of the data set had minimal impact on the performance of MC, further reinforcing its stability and reliability across various simulation scales. For CTGAN, while larger data sets did contribute to a slight reduction in KLD values, the effect was less pronounced compared to the impact of increasing the number of epochs. This suggests that for CTGAN, the duration of training is more critical than the volume of data to be simulated. However, longer training durations are computationally expensive, and if 50,000 epochs still result in high KLD values, it may not be practical to extend the training further.

The MC method performed well in this study scenario probably because it is based on a straightforward statistical approach. By sampling from the distributions (mean and covariance) of the original data, it simulates data that naturally aligns with the statistical properties of the real-world samples. This leads to a lower KLD. CTGAN while powerful, require large and diverse datasets to effectively learn the complex relationships in the data. With only 27 CO and 10 SO samples, the CTGAN model likely lacked sufficient data to fully capture these relationships, leading to higher KLD. This could be a possible explanation why the MC performed better than the CTGAN with the sample set that we have in the study.

MC simulation has been successfully applied in other studies that used comparable original sample sizes for the simulation of vegetable oils, further supporting our findings (Zhang et al., 2014, 2017a, 2017b). Although these studies did not compare MC with other simulation models nor detailed the specific implementation, the consistent results suggest the robustness of the MC approach in similar scenarios. In contrast, to the best of our knowledge, GAN has not yet been applied to the chemical composition of oils using conventional tabular datasets for simulating vegetable oil compositions, making direct comparisons with similar studies difficult. However, other GAN variants have been successfully employed with hyperspectral images to generate sufficient synthetic data for training models aimed at different objectives, such as predicting oil content in maize (using deep convolutional GAN) (Zhang et al., 2022) or polyunsaturated FA content in meat (using autoencoder-assisted GAN) (Cui et al., 2024). After multiple iterations, these GAN models were able to generate synthetic data that closely resembled experimental data (Zhang et al., 2022; Cui et al., 2024). Thus, the performance of GAN models depends significantly on the specific scenario, data type, and study objectives. Indeed its application as data simulation in food authenticity is still in its early stages (Deng et al., 2024).

The artificial pure oils simulated using MC were then used to calculate different levels of adulteration artificially using the weighted sum formula (Formula 2) as explained in the method section. Table 2 shows the number of the data simulated for each class which will be used for the training of the classification models.

**Table 2:** Number of simulated samples for each class.

Classes	Number of simulated samples
CO (100% pure CO)	1,000/10,000/100,000/1,000,000
SO (100% pure SO)	1,000/10,000/100,000/1,000,000
99:1 (Mixture of 99% CO and 1% SO)	1,000/10,000/100,000/1,000,000
97:3 (Mixture of 97% CO and 3% SO)	1,000/10,000/100,000/1,000,000
95:5 (Mixture of 95% CO and 5% SO)	1,000/10,000/100,000/1,000,000
93:7 (Mixture of 93% CO and 7% SO)	1,000/10,000/100,000/1,000,000
91:9 (Mixture of 91% CO and 9% SO)	1,000/10,000/100,000/1,000,000

### 3.3 Classification models

For the classification task, RF and NN models were employed, utilizing data simulated by MC and the weighted sum formula (Table 2). The data was split into 80% for training and 20% for testing. Hyperparameter tuning was performed for both classification models. Tuning the hyperparameters is a critical step before proceeding to the classification and it helps to reduce the overfitting of the classification model, which may happen when default hyperparameters are used (A Ilemobayo et al., 2024). The tuning of the hyperparameters was done for both NN and RF as described in section 2.5.2. The optimized hyperparameters for RF and NN are reported in Table S1 and Table S2, respectively.

After training the models on simulated data, their performance was first assessed using a separate set of simulated test data (section 3.3.1) and was validated later using small real-world dataset (section 3.3.2). Model performance was primarily evaluated using accuracy, which represents the proportion of correctly classified oil samples out of all predictions made by the model. In addition to accuracy as a global performance metric, also confusion matrices and classification reports were examined, which provide additional insights into model performance at the class level through metrics such as precision, recall, and F-score.

#### 3.3.1 Evaluation of the classification model performance using simulated data

Table 3 presents the classification accuracies of the RF and NN models for various quantities of simulated samples. The table highlights how the performance of each model scales as the amount of available data for training/testing grows, which is crucial for understanding their potential.

**Table 3:** Performances of Random Forest (RF) and Neural Network (NN) on simulated data (classification accuracies %).

Number of simulated samples per class	RF	NN
1,000	83.7%	92.0%
10,000	90.5%	92.0%
100,000	92.5%	93.0%
1,000,000	94.0%	93.0%

The RF model showed an improvement in classification accuracy as the number of simulated samples used to train/test the model increases. Starting with 1,000 samples per class, RF achieves an accuracy of 83.7%. This improves to 90.5% with 10,000 samples per class, and continue to rise, reaching 94% with 1,000,000 samples per class. The trend suggests that RF benefits from larger training datasets, which likely allows the model to capture more complex patterns and variability in the data. Another relevant enhancing factor is the use of hyperparameter tuning, which allows determining which hyperparameters are optimal for each dataset size. However, the accuracy increases from 100,000 samples per class (92.5%) to 1,000,000 samples per class (94%) were relatively modest, suggesting that the relevant patterns were possible captured by the algorithm. It is a quite natural behavior that algorithms don't show a linear improvement with increasing number of observations, but may increase faster at the beginning and slowly later (Domingos, 2012).

In contrast, the NN model showed a pattern which were less related to the sample size. At smaller dataset sizes, NN starts with a high accuracy of 92% with 1,000 samples, outperforming RF at this initial stage. However, as the dataset size increases, NN's performance stabilizes, at accuracy values of 92% for 10,000, and 93% for 100,000 and 1,000,000 samples per class. While further tuning of NN parameters for 100,000 samples onwards could potentially improve its accuracy, it becomes highly complex and computationally demanding, especially with larger datasets. Therefore, the observed performance difference between RF and NN can be mainly attributed to the intrinsic characteristics of the models but also to some extent of the tuning applied.

**Fig. 2:**
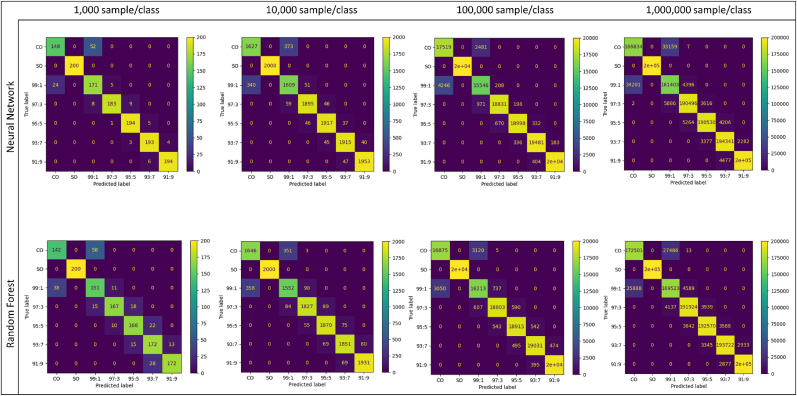
Confusion matrices showing the performances of Random Forest and Neural Network using test sets of different numbers of simulated samples.

For a more detailed visualization of the results, Fig. 2 shows the confusion matrices for the two classification models, RF and NN. Each column contains the confusion matrices for each size of the simulated data sets (1,000, 10,000, 100,000, and 1,000,000 samples/per class). The confusion matrix shows in detail the number of correct classified samples per class, which can be found on the diagonal, and the number of misclassified samples per class, located at the off-diagonals.

Both models consistently differentiate CO from SO across all dataset sizes, indicating that the fundamental distinction between the oils is well captured. However, the most frequent misclassifications occur between the 99:1 adulteration class and pure CO. This is not surprising, as the presence of only 1% SO in a mixture is difficult to distinguish from pure CO and may also fall within measurement error. Other misclassifications tend to occur between classes with minimal differences adulteration, such as 97:3 and 95:5 or 93:7 and 91:9. In other words, differences of ±2% SO will not be always recognized by both classification models. This is also supported by the values of precision, recall, and f1-score for which lower values for all the metrics where found for the challenging classifications (Fig. S3). Nevertheless, adulteration levels of 3% and higher are reliably identified by both models, and there are no misclassifications within a margin of ±4%. When comparing the models as the simulated dataset size increases, NN demonstrates reasonable classification performance at the 1,000 simulation, with more correct classifications in the confusion matrix (more samples in the diagonal) compared to RF at this early stage. However, as also indicated in Table 3, RF begins to gain in performance as the dataset size increases, showing improved accuracy and better class-level metrics, particularly for larger datasets. Even with 1% adulteration, the misclassifications remain reasonable for RF, as it classified around 14% of pure CO as 1% adulteration and around 13% of the 1% adulteration as pure CO, which is acceptable level of error.

#### 3.3.2 Evaluation of the classification model performance using real-world samples

Having developed and tested two classification model's RF and NN on simulated data, now the focus has shifted to assessing their performance using a smaller, real-world dataset of adulterated samples. This serves as an important validation step to ensure that the models, which were trained and tested on multivariate normal distributions (MC simulated data), can be effectively applied in real-world scenarios. A total of 33 adulterated CO samples were prepared, as detailed in the methods section. These represent approximately 2.4% (33 out of 27 CO × 10 SO × 5 adulteration levels) of all possible combinations. Table S3 provides details on the number of adulterated CO samples prepared for each level of adulteration.

When evaluating the models on real-world samples (Table 4), the observed accuracies were consistently lower than those achieved with the simulated data. However, in every case, they remained close or above 70%, which is reasonable considering the number of classes (seven), the possible variation of adulterations which may be not always exact at the assumed levels, the potential unobserved heterogeneities in real-world conditions and the small sample size of specific oils.

**Table 4:** Performance of trained Random Forest and Neural Network tested on real-world samples after being trained/tested on different numbers of simulated samples (classification accuracies %).

Number of simulations used to train/test the model	RF	NN
1,000	68.5%	69.0%
10,000	74.2%	73.0%
100,000	88.5%	81.0%
1,000,000	90.0%	74.0%

Considering the performances of RF, the differences between simulated and real-world data become smaller when the size of the simulated datasets becomes larger. The smallest distance of 4% is reached for the RF trained on one million samples of simulated data. This suggests that with larger datasets not only the performances of RF become better, but also the capacity of the model to generalize is enhanced, so that the risk of overfitting is reduced. The combination of both hyperparameter tuning and also using larger datasets for training yields enhances robustness for RF models. Considering the performances of NN, the accuracies were in most cases lower than the RF. The only exception was found for a smaller dataset (1,000 samples), where NN achieved a slight accuracy advantage over RF (with ca. 0.5% points higher). However, when the models were trained on larger datasets (10,000 and above), RF clearly outperformed NN. Therefore, the NN seems to be less well generalizable to real-world conditions as the RF. This behavior is often observed, when a model focuses too much on certain given data points than learning the overall pattern.

**Fig. 3:**
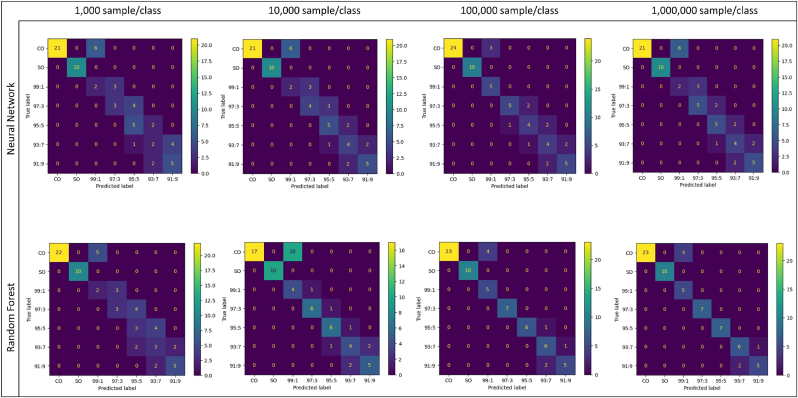
Confusion matrices of trained Random Forest and Neural Network models, tested on real-world samples after being trained/tested on different numbers of simulated samples.

Further insights into model performance are provided by the confusion matrices in Fig. 3 and the classification metrics in Fig. S4. Both models correctly classified pure oils, but minor misclassifications occur in the other classes, primarily between classes that were close in adulteration level. These misclassifications followed the same pattern as observed with the simulated data, with both models struggling more with classes that differ by only small amounts of adulteration. Notably, models trained with larger datasets tend to correctly classify more samples, particularly in the case of RF. As the number of collected pure oil samples was limited, the same CO and SO real-world samples used for estimating the underlying distributions for MC simulations, were also used as the basis for generating real-world adulterated samples. It is not expected that this has a large impact on the validation since the real-world samples were considered as random realizations of the underlying distribution. However, a positive effect on the model performance cannot be entirely excluded. Therefore, further independent evaluations are recommended to confirm the promising results reported here, providing a more robust validation.

When comparing the models in terms of practical application, both performed well; however, RF exhibited greater robustness, especially with larger datasets, and consistently achieved better classification accuracy, on both simulated and real-world samples. Additionally, KLD values (Table 1) indicate that datasets with 10,000 or more samples provided a closer fit to observed data than those with 1,000 samples, implying that the advantage of NN at 1,000 samples was not as relevant as the performance in the settings with more samples.

From the perspective of CO authenticity testing and possibly oil authenticity in general model interpretability is an important consideration. RF offers an advantage here, as its decision trees provide less complex, traceable decision-making processes, which can be crucial for regulatory purposes where transparency is required. In contrast, NN operates more like a "black box", which could make it less practical in situations requiring interpretability (Roβbach, 2018). Furthermore, RF models are computationally less demanding, requiring less time and fewer hardware resources for training and optimization compared to NN, which can be demanding when working with large datasets which seem to be needed to capture the observed data sufficiently. Although NN shows stable performance and has the ability to capture complex patterns, the comparable and often better accuracy of RF in our case, combined with its interpretability and computational efficiency, suggests that RF is the more suitable model for this particular scenario.

## 4 Conclusion

This study explored the integration of data simulation and machine learning techniques to address the challenge of detecting adulterated cactus seed oil (CO) in order to evaluate the limit of adulteration that can be detected without measuring a huge number of different mixtures. From a methodological perspective, Monte Carlo simulation proved to simulate more realistic oil composition data, in our case, compared to Conditional Tabular Generative Adversarial Network, enhancing model performance and reducing the need for large-scale sample collection. While Neural Networks showed potential in capturing complex patterns, Random Forests interpretability and practicality make it more suitable for this setting. By employing a Monte Carlo simulation with Random Forest classification, we achieved high accuracy (90%) in detecting adulteration of CO with refined sunflower oil, with detectable adulteration levels as low as 1%. As a proof-of-concept, the approach has exemplarily demonstrated its potential specifically for an adulteration of Moroccan CO with SO. It could be feasibly implemented as a routine protocol in quality control laboratories in order to detect adulterated CO and estimate the level of adulteration. The open-access Python-based methodology ensures that the models can be continuously updated with new data, making it adaptable for future studies. This methodology has significant implications for protecting the authenticity of CO, reinforcing consumer trust, and supporting Moroccan cooperatives. However, further evaluations in real-world settings will be necessary to refine its applicability. Future research should explore the scalability and robustness of the method for broader CO adulteration scenarios. This includes addressing mixtures involving various refined oils, incorporating larger and more diverse datasets, and enhancing the model's ability to handle missing values. From a methodological perspective, the proposed approach can be extended to other types of oils, as long as suitable data are utilized and the model is properly trained and tuned.

## CRediT authorship contribution statement

**Said El Harkaoui:** Conceptualization, Methodology, Investigation, Writing – original draft, Visualization, Project administration. **Cristina Ortiz Cruz:** Methodology, Software, Formal analysis, Writing – original draft, Visualization. **Aaron Roggenland:** Methodology, Software, Formal analysis, Writing – original draft, Visualization. **Micha Schneider:** Methodology, Data curation, Formal analysis, Writing – review & editing. **Sascha Rohn:** Validation, Data curation, Writing – review & editing, Supervision. **Stephan Drusch:** Validation, Data curation, Writing – review & editing, Supervision. **Bertrand Matthäus:** Conceptualization, Writing – review & editing, Supervision, Funding acquisition.

## Funding

This work was supported by funds of the 10.13039/501100005908Federal Ministry of Food and Agriculture 10.13039/501100005908BMEL based on a decision of the Parliament of the Federal Republic of Germany via the 10.13039/501100010473Federal Office for Agriculture and Food (10.13039/501100010771BLE) [support program: FKZ 2819DOKA03 and FKZ 28KIDA007].

## Declaration of competing interest

The authors declare that they have no known competing financial interests or personal relationships that could have appeared to influence the work reported in this paper.

## Data Availability

The data publications containing the data used in this article are referenced in Section 3.1 and are available in the OpenAgrar repository, along with their corresponding DOIs. The Python script that implements the entire methodology is openly accessible at the following link: https://github.com/kida4bmel/oil-adulteration.
