# Supplementary material for: Adulteration detection in cactus seed oil: Integrating analytical chemistry and machine learning approaches
Source: Curr Res Food Sci. 2025 Jan 22;10:100986. doi: 10.1016/j.crfs.2025.100986 (PMC11821398; doi:10.1016/j.crfs.2025.100986)
Supplement: Multimedia component 2 [file mmc2.docx]

**Adulteration Detection in Cactus Seed Oil: Integrating Analytical Chemistry and Machine Learning Approaches**

Said El Harkaoui ^a,b,c,*^, Cristina Ortiz Cruz ^dg^, Aaron Roggenland ^eg^, Micha Schneider ^fg^, Sascha Rohn ^b^, Stephan Drusch ^c^, and Bertrand Matthäus ^a^

^a^ Max Rubner-Institut, Federal Research Institute for Nutrition and Food, Department for Safety and Quality of Cereals, Schützenberg 12, 32756 Detmold, Germany.

^b^ Department of Food Chemistry and Analysis, Institute of Food Technology and Food Chemistry, Technische Universität Berlin, Berlin, Germany.

^c^ Department of Food Technology and Food Material Science, Institute of Food Technology and Food Chemistry, Technische Universität Berlin, Berlin, Germany.

^d^ Max Rubner-Institut, Federal Research Institute for Nutrition and Food, Zentralabteilung, Haid-und-Neu-Str. 9, 76131 Karlsruhe

^e^ Max Rubner-Institut, Federal Research Institute for Nutrition and Food, Zentralabteilung, Schützenberg 12, 32756 Detmold, Germany.

^f^ Johann Heinrich von Thünen Institute - Federal Research Institute for Rural Areas, Forestry and Fisheries, Bundesallee 50, 38116 Braunschweig

^g^ BMEL project KIDA, AI consultancy

^*^Corresponding author at Max Rubner-Institut, Federal Research Institute for Nutrition and Food, Department for Safety and Quality of Cereals, Schützenberg 12, 32756 Detmold, Germany

E-mail address: Said.Elharkaoui@mri.bund.de (Said El Harkaoui)


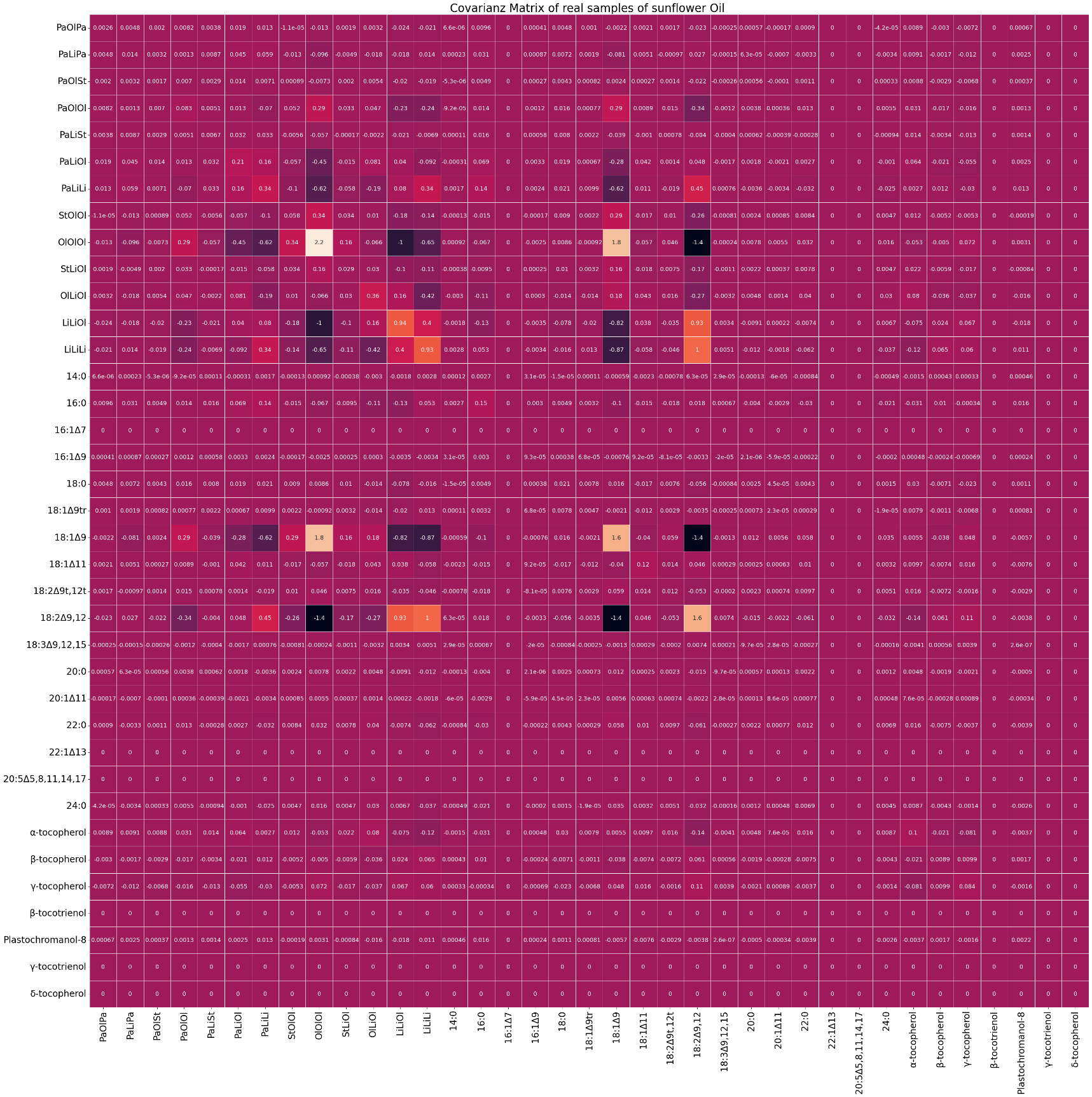
**Fig. S1.** Covariance matrix for the chemical composition of refined sunflower oil


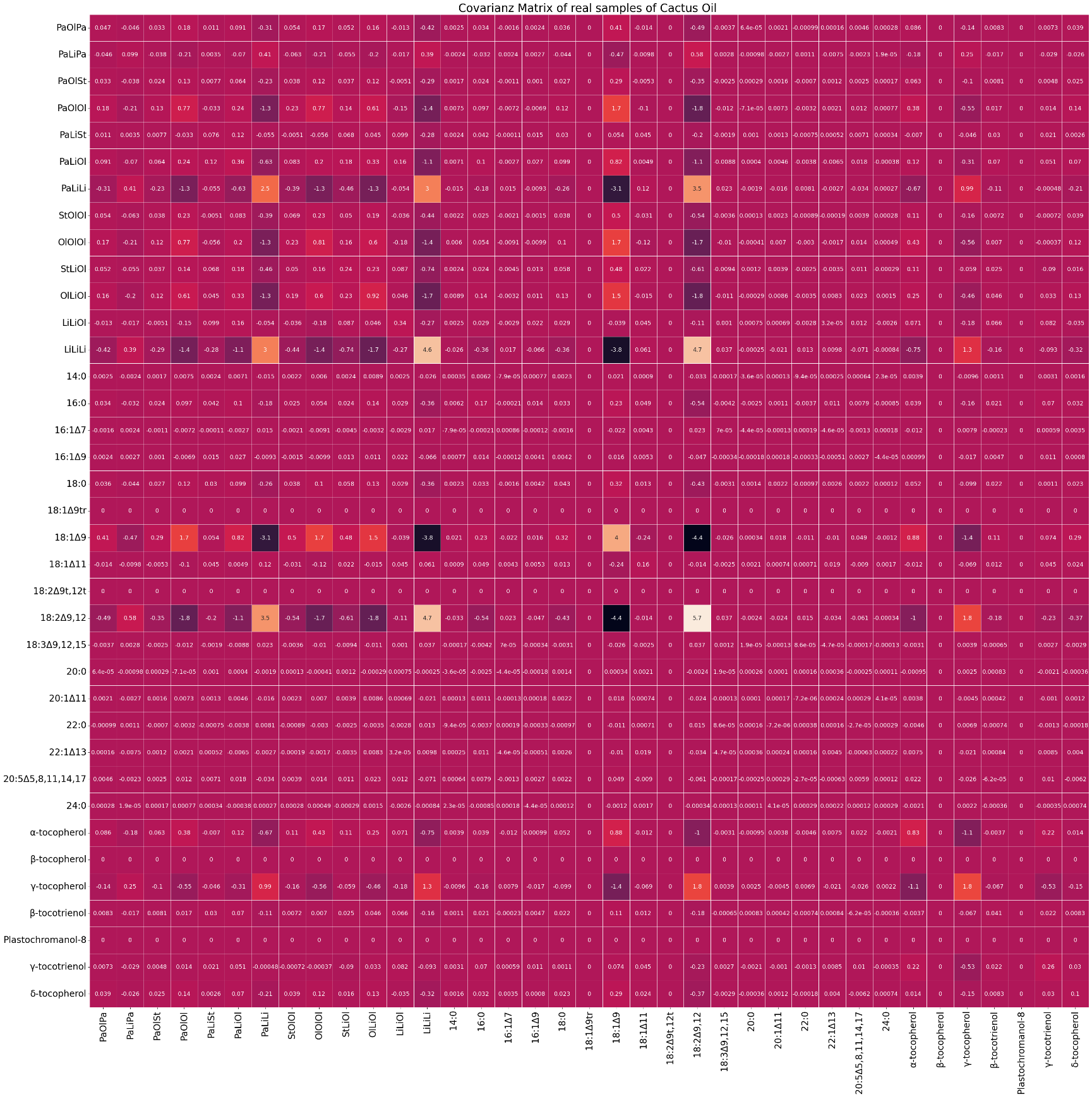
**Fig. S2.** Covariance matrix for the chemical composition of cactus seed oil

**Table S1**

Hyperparameter set for Random Forest (RF) models trained on data sets of different sizes.

| Model | max_depth | Bootstrap | min_samples_split | max_features | min_samples_leaf | n_estimators |
| --- | --- | --- | --- | --- | --- | --- |
| RF trained on 1,000 samples | 9 | True | 6 | All features | 3 | 1,500 |
| RF trained on 10,000 samples | 24 | True | 2 | All features | 9 | 500 |
| RF trained on 100,000 samples | 24 | True | 2 | All features | 9 | 500 |
| RF trained on 1,000,000 samples | 24 | True | 2 | All features | 9 | 100 |

The following values were used in the grid for tuning Random Forest: max_depth (values from 2 to 24), Bootstrap (True or False), min_samples_split (values from 2 to 8), max_features (all features or sqrt (square root of number of features), min_samples_leaf (values from 3 to 9), n_estimators (values from 100 to 1500). Note: For 1,000,000 simulations, the number of n_estimators were fixed at 100 and was not determined by grid search.

**Table S2**

Hyperparameter set for Neural Network (NN) models trained on data sets of different sizes.

| Model | Number of Hidden-Layers | Dimension for each hidden Layer | Batch_size |
| --- | --- | --- | --- |
| NN trained on 1,000 samples | 1 | Layer1: 91 | 16 |
| NN trained on 10,000 samples | 2 | Layer1: 51  Layer2: 69 | 18 |
| NN trained on 100,000 samples | 2 | Layer1: 51  Layer2: 69 | 18 |
| NN trained on 1,000,000 samples | 2 | Layer1: 51  Layer2: 69 | 18 |

The following values were used for the tuning: Number of Hidden-Layers (values of 1 to 3), Dimension for each hidden Layer (values between 32 and 128), Batch size (values between 16 and 64). Note: For 100,000 and 1,000,000 simulations, complete tuning was not feasible due to hardware limitations. Thus, the hyperparameters optimized for 10,000 simulations were reused.


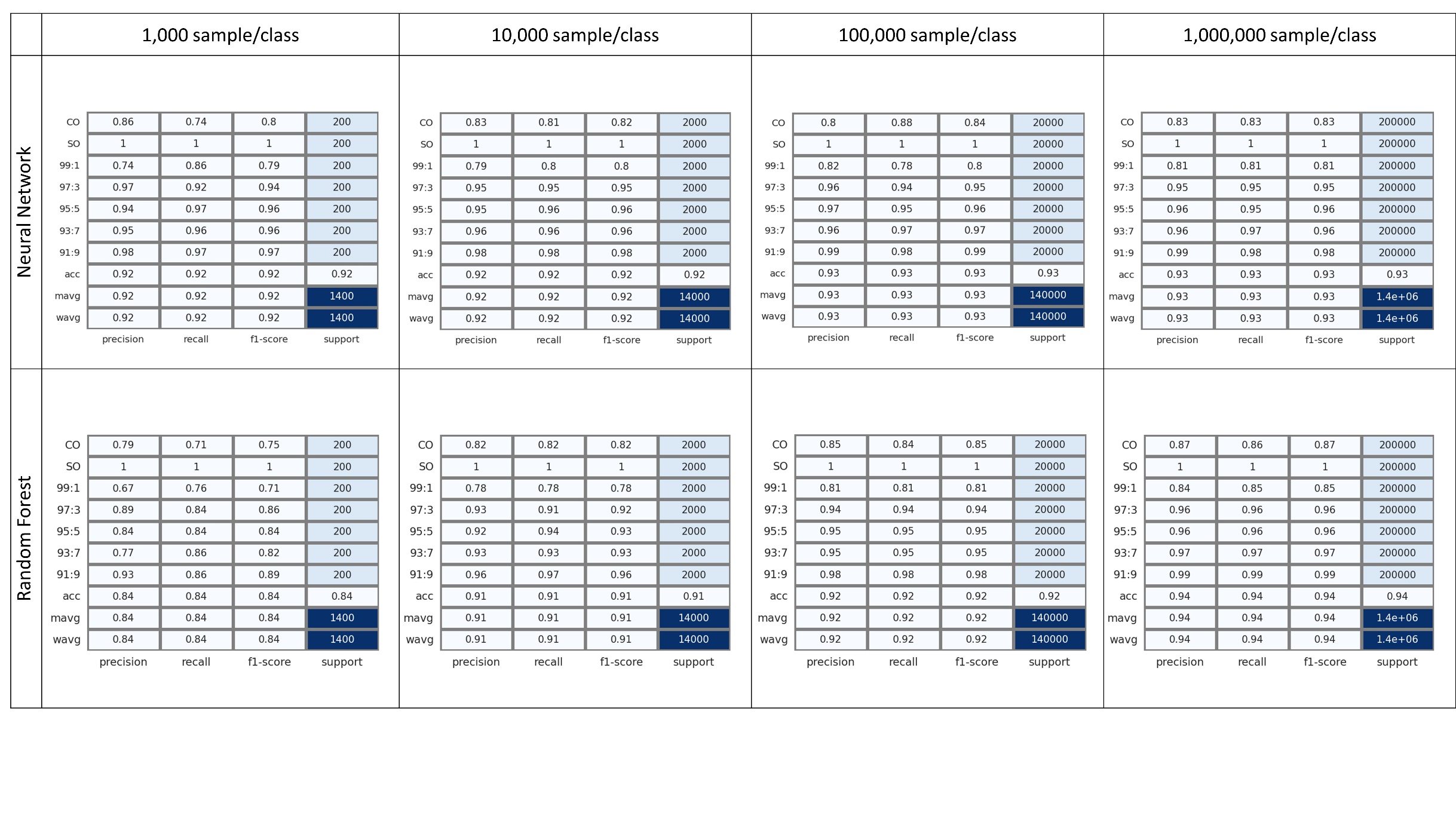
**Fig. S3.** Classification metrics on a class level for Random Forest and Neural Network using test sets of different simulation numbers. Accuracy (acc), Macro average (mavg), Weighted average (wavg).

**Table S3**

Number of real samples for each class.

| **Classes** | **Number of real samples** |
| --- | --- |
| CO (100% Cactus oil) | 27 |
| SO (100% Sunflower oil) | 10 |
| 99:1 (Mixture of 99% CO and 1% SO) | 5 |
| 97:3 (Mixture of 97% CO and 3% SO) | 7 |
| 95:5 (Mixture of 95% CO and 5% SO) | 7 |
| 93:7 (Mixture of 93% CO and 7% SO) | 7 |
| 91:9 (Mixture of 91% CO and 9% SO) | 7 |


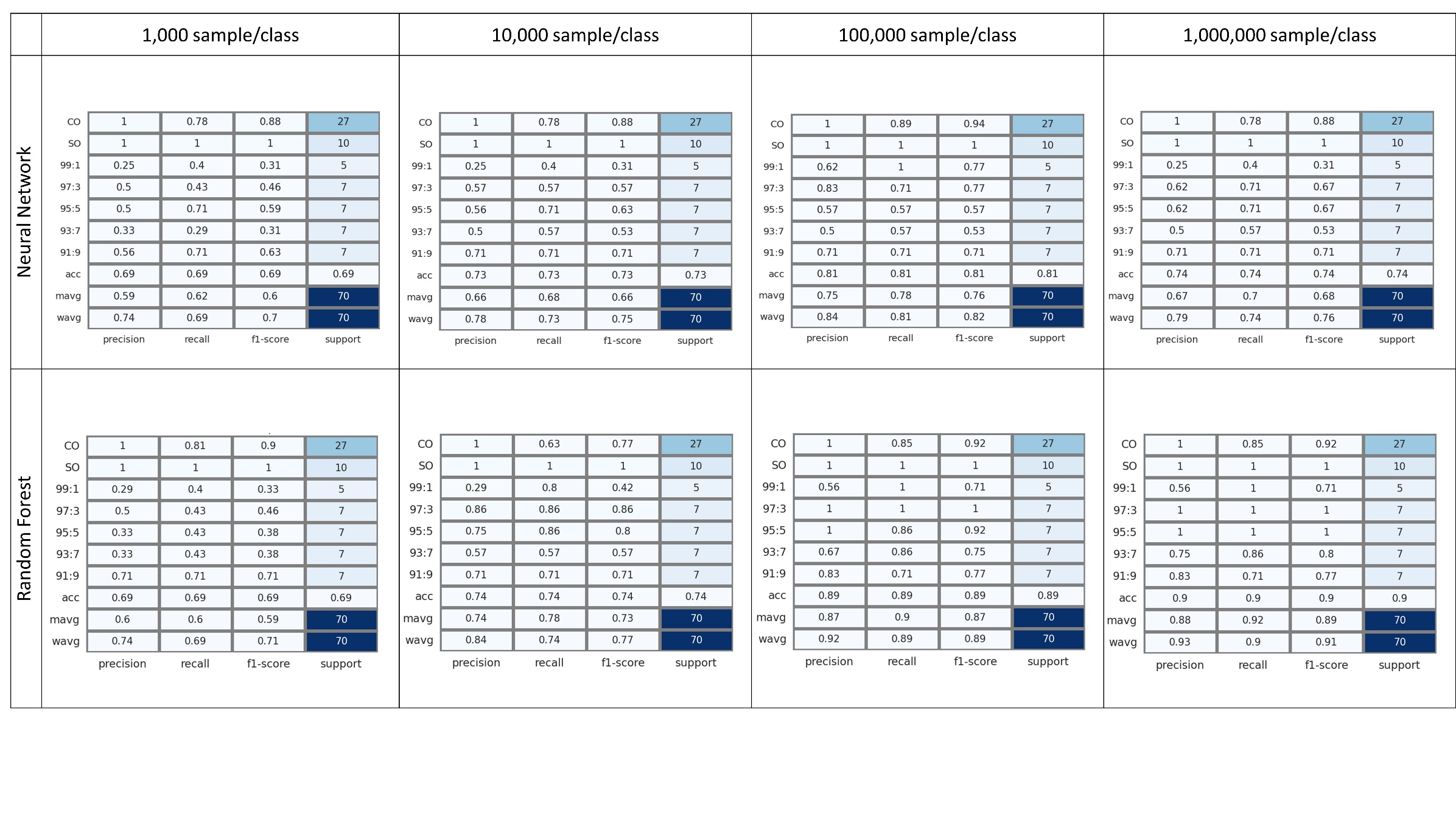
**Fig. S4.** Classification metrics on a class level for Random Forest and Neural Network tested on real-world samples after being trained on different numbers of simulated samples. Accuracy (acc), Macro average (mavg), Weighted average (wavg).
